# Editorial: Constructing the vascular or cardiac tissue and organoids: the combination of biomedicine and engineering

**DOI:** 10.3389/fcvm.2024.1371074

**Published:** 2024-02-16

**Authors:** Dayu Sun, Rajesh Katare, Palaniappan Sethu, Panke Cheng, Yonghong Fan

**Affiliations:** ^1^ Department of Anatomy, Third Military Medical University, Chongqing, China; ^2^ Department of Physiology, HeartOtago, Dunedin School of Medicine, University of Otago, Dunedin, New Zealand; ^3^ Division of Cardiovascular Disease, Department of Medicine and Department of Biomedical Engineering, University of Alabama at Birmingham, Birmingham, AL, United States; ^4^ Institute of Cardiovascular Diseases & Department of Cardiology, Sichuan Provincial People’s Hospital, School of Medicine, University of Electronic Science and Technology of China, Chengdu, China; ^5^ Laboratory of Basic Medicine, The General Hospital of Western Theater Command, Chengdu, China

**Keywords:** vessel, cardiac function, heart, bioengineering, tissue engineering, organoid, biomaterials, manufacturing

**Editorial on the Research Topic** Constructing the vascular or cardiac tissue and organoids: the combination of biomedicine and engineering

Cardiovascular diseases (CVD) are the most prevalent noncommunicable conditions and remain the leading cause of death worldwide (1, 2). Despite pharmacotherapy being the first-line treatment (3), surgical repair or replacement is recognized as an indispensable therapy for patients with severe CVD. Due to the shortage of donors and immune responses, bioengineered vascular tissue and cardiac tissue are promising strategies to solve these problems (4, 5).

Tissue engineering utilizes the principles of engineering and life sciences to the generation of biological substitutes (6). Its key elements consist of cell sources, scaffolds, biochemical cues, and fabrication techniques (7). With cutting-edge technology in biomedicine and engineering, such as organoids (8), microfludics (9), and organ-on-a-chip (10), scientists are attempting to biofabricate functional and physiologically-relevant tissues, and even organs.

Based on this topic, the compilation includes three review articles and one original research article, exploring the progress in bioengineered vascular tissue and bioengineered cardiac tissue.

## Bioengineered vascular tissue

Traditional vascular grafts include synthetic conduits, xenografts of animal origin, cadaveric allografts, and autografts from patients' own bodies. They have limited supplies and frequent complications due to mechanical stress, inflammatory responses, and inconsistent remodeling. In comparison, bioengineered vascular tissue has the potential to overcome the issues mentioned above, be remodeled by the host into native tissue, and even grow with young patients (11).

The review by Leal et al. encompasses the development, manufacturing techniques, fabricating materials, *in vivo* animal studies, and clinical trials of small-caliber vascular grafts. From their perspective of cardiac surgeons, the cell-free scaffold-based small-diameter TEVG made from biocompatible polymers would meet the significant clinical demand. A variety of materials' unique features and properties are classified, and a combination of these polymers, as well as scaffold modifications with biomolecules and functional cells, are suggested. This might contribute to creating ideal and practical small-caliber vascular grafts.

Campanile et al. summarize the niche structure, function, and *in vitro* model of the bone marrow vasculature, and compare cellular mobilization and homing in the Bone Marrow via its vasculature in both cardiovascular disease and cancer. The researchers propose that a more efficient *in vitro* model should incorporate a monolayer of endothelial cells within a fluid flow environment, along with perivascular cells, of human origin and derived from bone marrow.

## Bioengineered cardiac tissue

Cardiac tissue engineering has been a major focus of the tissue engineering field. Since the adult heart lacks the ability to generate, implantation of cells and bioengineered cardiac patches is employed to repair the damaged myocardium (12). Progress in fabrication techniques, tissue maturation, vascularization and perfusion, and high-throughput platforms will propel bioengineered cardiac tissue toward true clinical and industrial application (13).

Birla summarizes the advancements in ventricle tissue engineering and identifies four main biofabrication strategies for bioengineering ventricles: bioprinting, pull-spinning, utilizing balloon catheters, and utilizing custom molds. In addition to fabrication technology, further progress in biomaterials, cell sourcing, and bioreactor technology would expedite the advancement of this field and bridge the functional gap between bioengineered and human ventricles.

Reyat et al. have developed a method to create vascularized and chamber-specific cardiac microtissues by combining atrial or ventricular cardiomyocytes from hiPSC with vascular sprouts from human blood vessel organoids. The gene expression signatures, architectural structure, and electrophysiological properties of the microtissues are comparable to those of *in vivo*-derived cardiac tissues. Pro-fibrotic stimulation recapitulated the features of cardiac fibrosis. However, the phenotype can be reversed by the receptor inhibitor, indicating the potential of cardiac microtissues in disease modelling and pharmacological screening.

## Conclusion

These articles and reviews on the topic show that it is very promising to treat cardiovascular disease with bioengineered vascular or cardiac tissues and organoids. The advancements in both biomedicine and engineering, as well as their further collaboration, would lead to improved treatments for cardiovascular disease.
